# Single Amino Acid Polymorphisms of Pertussis Toxin Subunit S2 (PtxB) Affect Protein Function

**DOI:** 10.1371/journal.pone.0137379

**Published:** 2015-09-16

**Authors:** Scott H. Millen, Mineo Watanabe, Eiji Komatsu, Fuminori Yamaguchi, Yuki Nagasawa, Eri Suzuki, Haleigh Monaco, Alison A. Weiss

**Affiliations:** 1 Department of Molecular Genetics, Biochemistry, and Microbiology, University of Cincinnati, Cincinnati, Ohio, United States of America; 2 Graduate School of Infection Control Sciences, Kitasato University, Minato-ku, Tokyo, Japan; Centre National de la Recherche Scientifique, Aix-Marseille Universitté, FRANCE

## Abstract

Whooping cough due to *Bordetella pertussis* is increasing in incidence, in part due to accumulation of mutations which increase bacterial fitness in highly vaccinated populations. Polymorphisms in the pertussis toxin, *ptxA* and *ptxB* genes, and the pertactin, *prn* genes of clinical isolates of *Bordetella pertussis* collected in Cincinnati from 1989 through 2005 were examined. While the *ptxA* and *prn* genotypes were variable, all 48 strains had the *ptxB2* genotype; *ptxB1* encodes glycine at amino acid 18 of the S2 subunit of pertussis toxin, while *ptxB2* encodes serine. We investigated antigenic and functional differences of PtxB1 and PtxB2. The S2 protein was not very immunogenic. Only a few vaccinated or individuals infected with *B*. *pertussis* developed antibody responses to the S2 subunit, and these sera recognized both polymorphic forms equally well. Amino acid 18 of S2 is in a glycan binding domain, and the PtxB forms displayed differences in receptor recognition and toxicity. PtxB1 bound better to the glycoprotein, fetuin, and Jurkat T cells *in vitro*, but the two forms were equally effective at promoting CHO cell clustering. To investigate *in vivo* activity of Ptx, one μg of Ptx was administered to DDY mice and blood was collected on 4 days after injection. PtxB2 was more effective at promoting lymphocytosis in mice.

## Introduction

Pertussis is a highly contagious disease caused by *Bordetella pertussis*. The introduction of the whole cell pertussis vaccines in the 1940s caused a dramatic decrease in the number of cases and deaths due to pertussis [1]. Due to vaccine safety concerns, in the early 1990s the United States switched from using whole cell pertussis vaccines to acellular pertussis vaccines. While formulations differ, purified component acellular vaccines contain at most five different antigens; toxoided pertussis toxin (Ptx), filamentous hemagglutinin (FHA), pertactin (Prn), and two antigenic forms of fimbriae (Fim). Over the past 25 years, the number of reported cases of pertussis in the United States has been increasing even though vaccination rates have remained very high, making pertussis the only vaccine-preventable disease displaying a dramatic increase in incidence in fully vaccinated populations. Fully immunized children aged 7–10 years now account for most of the cases in the U.S. [2]. When the source of the initial vaccine (i.e. priming vaccine) was examined, the reported rate of pertussis was significantly lower in the children who were primed with whole cell pertussis vaccine compared to acellular vaccine, regardless of whether they had completed the full immunization series, or had received the acellular adolescent booster dose [2,3]. Taken together, these results strongly suggest that the current pertussis vaccines fail to confer lasting protection and that acellular vaccines are less effective than whole cell vaccines.

Polymorphisms in the genes for the vaccine antigens Ptx and Prn, have been observed with increasing frequency, and the amino acid sequence in circulating strains can differ from the vaccine antigens [4–6], for example, the *ptxA1* form of pertussis toxin and *prn2* form of pertactin were dominant genotypes in recent clinical isolates, whereas the vaccines were formulated from strains expressing the *ptxA2* and the *prn1* alleles. These alleles do not appear to alter protein function; the *ptxA* polymorphisms did not alter virulence [7], and isogenic strains expressing different alleles of *prn* colonized the lungs of mice equally well [8]. It has been proposed that vaccine antigens induce antibodies that are less effective against the new polymorphic forms, allowing the bacteria to escape from vaccine protection [9–11]. Mouse challenge studies with isogenic strains [8] lend support to this hypothesis.

It is widely believed that Ptx is the most critical vaccine antigen for preventing serious, life-threatening disease, while an immune response to other antigens aids in preventing bacterial colonization. Ptx is a member of the AB_5_ family of bacterial toxins. The single enzymatically active (or A) subunit (called PtxA or S1) is an ADP-ribosyltransferase which targets the α-subunit of some GTP-binding proteins. The binding (or B) subunit, is a pentamer, with 4 different subunits, called PtxB (or S2), PtxE (or S3), PtxC (or S4), and PtxD (or S5), in the ratio 1:1:2:1. The five B subunits are arranged in a ring structure and the A-subunit associates via the pore of the pentameric ring. The B-pentamer is required for the targeting and cytosolic entry of S1 into mammalian cells.

**Fig 1:**
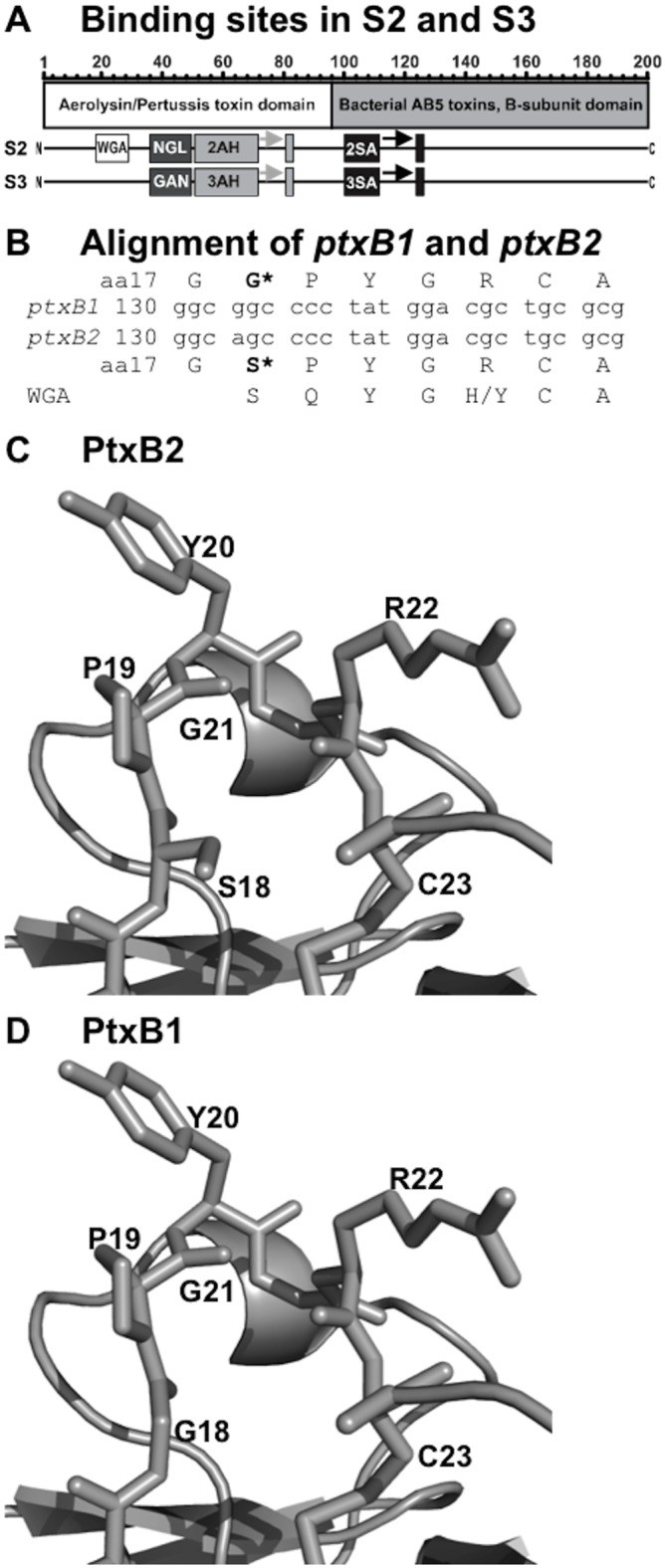
Glycan binding sites in Ptx. A. Putative glycan binding sites are indicated for the S2 and S3 subunits: wheat germ agglutinin homologous site (WGA), neutral glycolipid binding site on S2 (NGL), ganglioside binding site on S3 (GAN), aerolysin-homologous sites on S2 and S3 (2AH and 3AH), and sialic acid binding sites (2SA and 3SA). Discontinuous domains are denoted by arrows. B. Sequence comparison of the *ptxB1*and *ptxB2* alleles, and the WGA homology consensus sequence. C. Ptx crystal structure (1PTO, PtxB2) cartoon and stick representation focused on the polymorphism site. D. An in silico model of the PtxB1 phenotype with glycine at position 45 based on the 1PTO structure.

While the B-pentamer has four distinct subunits, all of the amino acid residues involved in mammalian receptor binding map to the S2 and S3 subunits [12]. S2 and S3 contain two domains (Fig 1A). The C-terminal domain (amino acids 90–200) is called the Bacterial AB_5_ toxins B-subunit domain. It possesses a small, well defined sialic acid (SA) binding site, while the rest of the C-terminus forms the protein fold necessary for subunit polymerization. The N-terminal domain (amino acids 1–89) is called the Aerolysin/Pertussis toxin domain. The N-terminal binding domain is less well defined, and may form one large binding domain that recognizes glycan chains. While, S2 and S3 share 71% amino acid identity, they display different binding preferences, largely mediated by differences in the N-terminal region [12].

Interestingly, the B-pentamer mediates toxic activities independent of the enzymatic activity of S1, a relatively new concept in the A-B model of protein toxin biology. B-pentamer activities include mitogenicity and T cell activation [13,14]. The molecular basis for the B-pentamer activity has been best studied using the human Jurkat T cell line. The B-pentamer binds glycoproteins from the T cell receptor (TCR) complex via their glycans, and promotes clustering, which in turn leads to antigen-independent activation the TCR [14,15]. This is the same mechanism plant lectins use to activate the TCR [15]. Receptor clustering requires one molecule to possess at least two glycan binding sites. The individual S2 and S3 subunits can promote TCR activation, but not if one of the sites has been mutated [12]. It is important to note that the enzymatic activity of pertussis toxin acts within the mammalian cytosol and the lectin-like activity of the B-pentamer acts extracellularly, via activation of cell-surface receptors. Thus, receptor engagement at the cell-surface by the Ptx B-pentamer can have two outcomes. One outcome can be engagement of receptors that promote toxin internalization, allowing the enzymatic domain to access its cytoplasmic target, while the other outcome is clustering of receptors at the cell-surface, promoting activation of their cell-signaling program. The relative importance of activation of cell-surface receptors versus inactivation of cytoplasmic GTP-binding proteins in the human disease process is currently not known, although receptor clustering requires much higher concentrations of toxin [14].

We investigated polymorphisms of *ptxA*, *ptxB*, and *prn* in clinical isolates collected in the Cincinnati area from 1989 through 2005. While multiple *ptxA* and *prn* alleles were observed, interestingly, only a single *ptxB* (*ptxB2*) allele was seen in all of the isolates. While the *ptxA* and *prn* alleles result in altered antigenicity, but are not known to alter protein function, the *ptxB* alleles change the amino acid sequence of a host receptor binding site within the S2 subunit (Fig 1). The nucleotide difference results in a coding change from glycine to serine in the S2 subunit (Fig 1B). Modeling of this amino acid change on the available PtxB2 crystal structure [16]demonstrates that the PtxB1 form would have a deeper and more open cleft between amino acids 18/19 and amino acids 22/23 than PtxB2 (Fig 1C and 1D). In this study, while we found no evidence for antigenic differences between the PtxB variants; these variants differ in toxin function as measured by leukocyte promoting activity in DDY mice.

## Materials and Methods

### Bacterial Strains

A total of 48 clinical isolates of *Bordetella pertussis* (Table 1) were obtained from 1989 through 1993, and 2005 at the Cincinnati Children’s Hospital Medical Center (Cincinnati, OH) [8,17,18]. *B*. *pertussis* strain Tohama I, which is a widely used vaccine strain, was used as a reference strain [19]. Strain CVI, an isogenic mutant described previously [8], and a Japanese clinical isolate KF-1, producing Ptx with PtxA1 and PtxB1 subunit and that with PtxA1 and PtxB2 respectively, were used in this study. Other reference strains are listed in Table 2 [20–24]. The amino acid sequences for PtxC-E were identical for these isolates. For routine propagation, *B*. *pertussis* was grown on Bordet-Gengou agar (BD Biosciences, Sparks, MD) supplemented with 1% glycerol and 15% defibrinated sheep blood (BG agar) at 36°C for 4 days. The isolates were stored at -80°C.

**Table 1:** Characteristics of clinical isolates from Cincinnati.

Strain	Year	*prn*	*ptxA*	*ptxB*
89-008-01	1989	1	2	2
89-002-01	1989	2	1	2
89-029-01	1989	1	2	2
89-046-01	1989	1	1	2
90-018-01	1990	2	1	2
90-019-01	1990	9	1	2
90-020-01	1990	2	1	2
90-021-01	1990	1	1	2
90-023-01	1990	1	2	2
90-031-01	1990	1	2	2
90-039-01	1990	1	2	2
90-040-01	1990	2	1	2
90-041-sm	1990	1	2	2
90–044	1990	1	2	2
90–048	1990	1	2	2
91–022	1991	2	1	2
92–035	1992	1	1	2
92–039	1992	1	2	2
93-025-01	1993	2	1	2
93-039-01	1993	2	1	2
93-042-01	1993	2	1	2
93-060-01	1993	1	2	2
93-075-01	1993	1	2	2
93-089-01	1993	2	1	2
93-098-01	1993	1	2	2
93-099-01	1993	1	2	2
93-106-01	1993	2	1	2
93-109-01	1993	2	1	2
CCHMC1 to CCHMC20	2005	2	1	2

**Table 2:** *PtxB* alleles in clinical isolates and vaccine strains.

Strain	Year, Country	Vaccine	*prn*	*ptxP*	*ptxA*	*ptxB*	reference
CVI	An isogenic mutant of Tohama I		1	1	1	1	[8]
KF1	2010, Japan	Japanese DTaP	2	3	2	2	This study
Tohama I	1952, Japan	GlaxoSmithKline(Infanrix, Boostrix)Lederle/Takeda adsorbed vaccine (Tetramune)	1	1	2	1	[19,25]
BP165	~1950, USA		NA^a^	1	2	2	[28]
L517	2006, Australia		1 with silent SNP	NA	1	2	[21]
B1831	1999–2000, Netherlands		2	3	1	2	[4,38]
B1834	1999–2000, Netherlands		2	1	1	2	[4,38]
B1920	1999–2000, Netherlands		2	1	1	2	[4,38]
CS	1952, China	Acellular vaccine	1	1	2	1	[22]
3779	1950s,USA	Eli Lilly vaccine	NA	1	2	2	[23,24]
10536	~1947,Unknown	Connaught whole cell vaccine—TriHIBit™Sanofi Pasteur (Daptacel, Adacel)	1	NA	2	2	NCBIaccession A13359

^a^NA, sequence information is not available.

### Sequencing

The clinical isolates were determined for polymorphism in *ptxA*, *prn*, and *ptxB*. DNA sequencing and alleles of *ptxA* and *prn* were performed as described by Mooi *et al* [4]. We confirmed Tohama I had *ptxB1* and *prn1*. Amplification of the region containing whole *ptxB* gene of each strain were performed using PtxXB-F (gcatcgcgtattcgttctagac) and PtxXB-R (tatgccgagcacggacaa), using PrimeSTAR HS DNA polymerase with GC buffer (Takara Bio, Ohtsu, Japan). After initial denaturation at 98°C for 2 min, the reaction was performed for 30 cycles at 98°C for 10 s, 60°C for 15 s and 68°C for 1.5 min followed by 5 min final extension at 68°C. The PCR products were sequenced with same primers.

### Purification of Ptx

**Fig 2:**
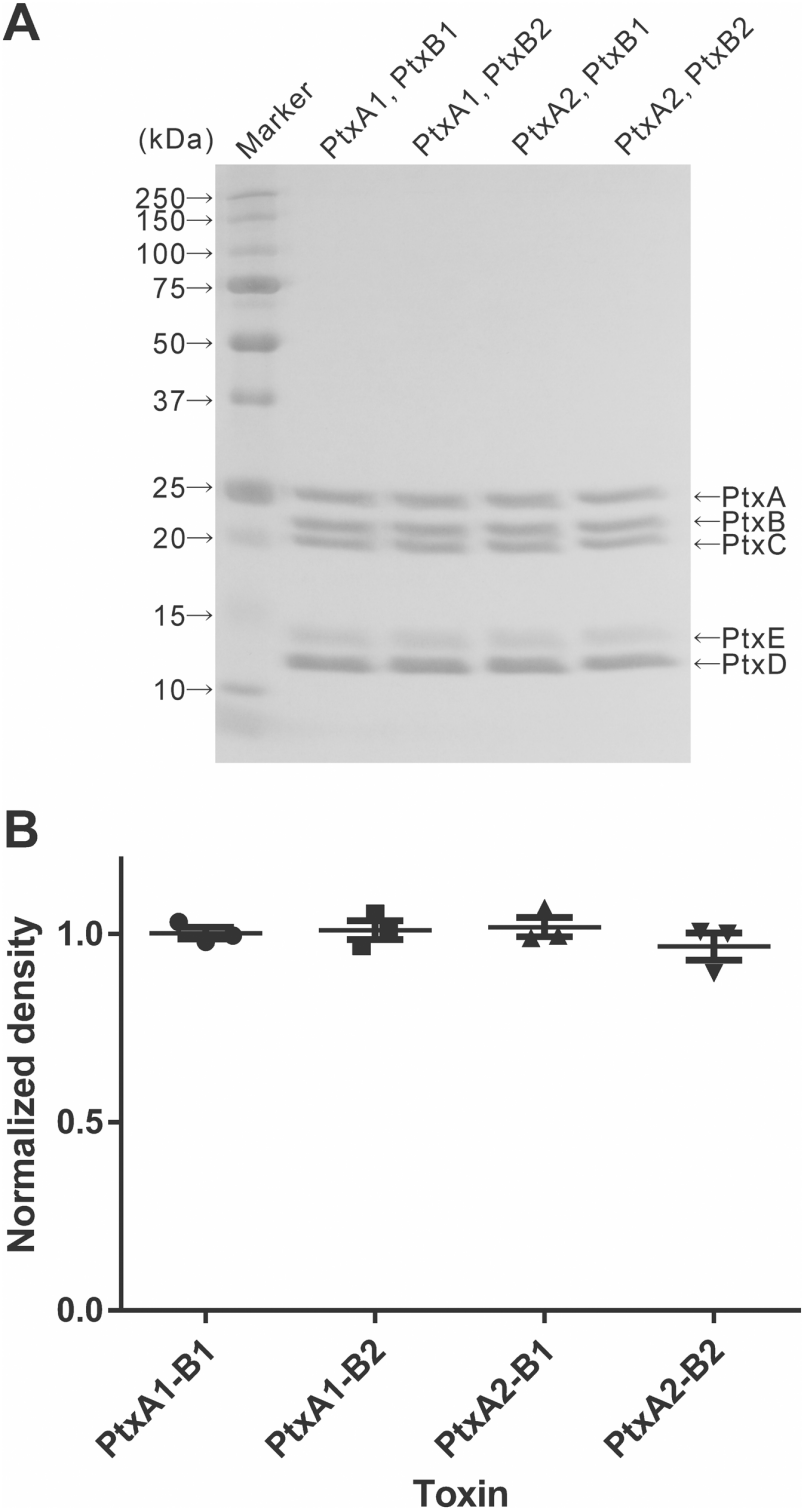
Purity of Ptx preparations. **A.** SDS-PAGE analysis of the four pertussis toxin variants. Purified toxin was loaded into each lane and separated electrophoretically on a 12% acrylamide gel. Representative example of three independent gels is shown. B. Densitometry to determine total protein amount was performed with ImageJ ver 1.47 software (http://imageJ.nih.gov/ij)), and individual values normalized to the mean are plotted., The standard deviation is 0.8%, and no statistical difference was determined for the different preparations.

*B*. *pertussis* strain Tohama I (producing Ptx with PtxA2 and PtxB1), and, CCHMC1 (producing Ptx with PtxA1 and PtxB2), CVI (producing Ptx with PtxA1 and PtxB1), and KF-1 (producing Ptx with PtxA2 and PtxB2), which were grown on BG agar at 36°C for 4 days were used to inoculate modified Stainer–Scholte medium containing 2.8% casamino acid. After the incubation for 5 days at 36°C as a static liquid culture, the cultures were centrifuged (4500×g, 30 min, 4°C) and filtered with 0.22 μm PVDF membrane to remove any remaining bacteria. The supernatant fluid was used for purification of Ptx as described previously [25–27]. In detail, the culture supernatant was applied to an AF-heparin Toyopearl 650M (Tosoh, Tokyo, Japan) column to remove filamentous hemagglutinin. The pH of the flow-through was adjusted to 6.0 by the addition of HCl. Affi-Gel blue resin (Bio-Rad, Hercules) was added to the fraction and incubated for 48 h at 4°C. The Affi-gel blue resin was washed with 0.25 M phosphate buffer, pH 6.0 and 0.05 M Tris–HCl buffer, pH 7.4 (buffer A). Proteins bound to the gel were eluted with buffer A containing 0.75 M MgCl2. Ptx-containing fractions were pooled and then dialyzed against buffer A containing 0.5 M NaCl at 4°C. After dialysis, the fraction was applied to a fetuin-conjugated Toyopearl 650M column that was prepared by the method recommended by the manufacturer (AF-Tresyl Toyopearl 650M, Tosoh; fetuin, Sigma). The column was washed with: buffer A containing 0.5 M NaCl; buffer A containing 1 M NaCl; buffer A containing 1 M NaCl and 2 M urea; and buffer A containing 0.5 M NaCl, in this order. Proteins bound to the column were eluted with 50 mM diethanolamine containing 0.5 M NaCl. Ptx containing-fraction was pooled as a final preparation. All of the preparations contained 0.063 endotoxin units/microgram of toxin. Purity was assessed by separating 5 μg by sodium dodecyl sulfate polyacrylamide gel electrophoresis using a 12% gel, followed by staining with BioSafe Coomassiee (BioRad). Only the five bands corresponding to the Ptx S1–S5 subunits were visible (Fig 2A), indicating purity. As noted in previous studies [28], the individual peptides of pertussis toxin do not stain uniformly; the S5 subunit is difficult to stain and it migrates slower than the S4 subunit, which has a higher molecular weight. Densitometry was performed on the stained gels and the values normalized to the mean are plotted (Fig 2B). The four preparations are not statistically different in total protein. Together these results indicate that the toxin preparations are pure, and that each of the four preparations is identical with respect the relative abundance of the five subunits.

### Purification of Recombinant Dimers

Construction and purification of S2(PtxB1)S4his and S3S4his expression constructs were described in previous studies [12]. The S2(PtxB2) expression construct was created by site-directed mutagenesis of the S2(PtxB1) expression construct. Briefly, recombinant Ptx subunits were engineered to contain *E*. *coli* N-terminal Sec-secretion signals. Signal peptides were cleaved and folding, disulfide bond formation and dimerization occurred in the periplasm. Dimers were purified from *E*. *coli* cell lysates using the His-tag, which was only expressed on PtxD, while the native mature amino acid sequence was present in the PtxB and PtxC proteins. Only two bands (corresponding to the sizes expected for PtxB and PtxD, or PtxC and PtxD) were seen by SDS-PAGE for the purified dimers.

### Glycan Binding of Recombinant S2(PtxB1)S4his, S2(PtxB2)S4his, and S3S4his Dimers to Fetuin and Asialofetuin

Fetuin or asialofetuin (lacking sialic acid) purchased from Sigma-Aldrich (St. Louis, MO) was suspended in PBS at 1 mg/mL and biotinylated at a 1:1 molar ratio using EZ-Link Sulfo-NHS-LC-Biotin (Thermo Scientific, Rockford, IL). To ensure equal loading, biotinylated fetuin or asialofetuin was loaded onto streptavidin-coated optical biosensors (Pall ForteBio Corp., Menlo Park, CA) and real-time binding was monitored by reflectometric interference spectroscopy using a ForteBio Octet. The loading step was stopped when the reflectometric interference shift reached 0.8 nm. The association and dissociation of recombinant dimers (716 nM, 25°C, 1000 rpm) was quantified by reflectometric interference spectroscopy.

### Binding of Ptx Dimers to the Human Jurkat T Cell Line

Recombinant dimers (200 nM) were incubated with Jurkat cells (10^6^) for 1 h at 4°C. The cells were washed and incubated with primary (anti-His monoclonal antibody) and secondary (FITC anti-mouse) antibodies. Fluorescence was measured by flow cytometry.

### Toxicity Assays

Activity of purified Ptx holotoxin was measured by the Chinese hamster ovary (CHO) cell-clustering assay described by Gillenius *et al* [29]. In detail, samples were serially diluted in 96-well flat bottom plates (100 μl per well) with Ham’s F-12 medium with 10% FCS, followed by addition of 100 μl of CHO cell suspension in same medium to each well. The cells were incubated for 24 to 48 h under 5% CO_2_ at 37°C, and the Ptx activity was expressed as the minimum amount of Ptx in well that induced cell clustering.

Lymphocytosis promoting activity was assessed by the method previously described by Morse and Morse [30]. All mice were housed in Animal center in Kitasato University. Mice were allowed free access to water and diet in a 12-hour light/dark cycle with room temperature at 23±2°C and 55±10% humidity. All cages filled with wood shavings. Ten to 18 animals were used per treatment. Sample size was calculated with the LaMorte’s power calculations spreadsheet (Boston University). One microgram of Ptx in 0.1 ml of PBS was injected intravenously via a lateral tail vein of 5-week-old specific pathogen free (SPF) female DDY mice (Japan SLC, Hamamatsu). Four days after the injection, blood was obtained from the tail under inhalation anesthesia (3% Isoflurane mixed with air). The blood was collected in blood collection tubes containing EDTA, and lymphocyte count was performed with a blood cell counter by KAC (Kyoto, Japan).

### Antigenicity of Pertussis Toxin Holotoxin by ELISA

Wells of 96-well microtiter plates were coated with 100 μL of purified Ptx holotoxin in 50 mM carbonate buffer, pH 9.6, for 16 h at room temperature. Wells were washed with washing buffer (PBS with 0.05% Tween 20), and incubated with 100 μL *B*. *pertussis* anti-serum (JNIH-12; NIBSC, Herfordshire, UK) diluted to 20 ELISA unit/mL in incubation buffer (PBS with 10% non-fat dried milk and 0.05% Tween 20) for 2 h at 37°C. Secondary alkaline phosphatase-conjugated goat anti-mouse IgG, diluted 1:5,000 in incubation buffer was added, and incubated for 2 h at 37°C. The wells were washed, and incubated with 200 μL of substrate (FAST-pNPP kit; Sigma, St. Louis, MO) for 30 min at room temperature, followed by addition of 25 μL of 12% NaOH. The optical density at 405 nm (OD_405_) was measured.

### Antigenicity of S2/S4 Heterodimer

Vaccinated individuals received the experimental acellular Amvax, Biocine or CLL-4 vaccines [31,32], or the three-component GSK vaccine used in the APERT acellular pertussis vaccine trial [13,33–35]. Convalescent sera were obtained from culture-positive children [28]. Values for ELISA units for Ptx IgG, neutralization of CHO cell clustering or neutralization of mitogenicity to peripheral blood mononuclear cells (PBMC) were reported previously [13,32,35,36]. All serum samples were stored frozen.

Antibody responses to the S2(PtxB1)S4his and S2(PtxB2)S4his heterodimers were determined using reflectometric interference spectroscopy. To eliminate non-specific binding due to salts, the serum samples were diluted 1 to 100 in PBS pH 7.4 and further purified by buffer exchange into PBS pH 7.4 using Zeba Spin Desalting Columns, 7K molecular weight cut-off (Thermo Scientific). HIS2 biosensor tips coated with poly-histidine antibody were loaded with purified heterodimers to give approximately equal loading (0.35 to 0.73 response units). The tips were blocked by incubation with 2% BSA in PBS. Equilibrium baseline values were taken in PBS. Antibody association was followed for 2000 seconds, and dissociation in PBS was followed for 1500 seconds. Samples with final dissociation values at baseline were considered negative and were not characterized further. Samples with final dissociation values above baseline were considered to be positive.

### Statistical Analysis

The statistical significance of differences between results for different samples was examined by appropriate statistical tests with GraphPad Prism software version 5 (GraphPad Software, La Jolla, CA.). Significance was accepted at *P* < 0.05.

### Ethics Statement

The animal studies were approved by the Animal Research Committee of Kitasato University (Tokyo, Japan) and conducted according to the guideline of the Ministry of Education, Culture, Sports, Science, and Technology. Human samples were collected with written informed consent under IRB protocols at Baylor University and Cincinnati Children’s Hospital Research Foundation. The de-identified samples were provided for characterization of immune responses to *B*. *pertussis* as permitted by the IRB protocols under which they were collected. Studies with these de-identified samples were approved by the University of Cincinnati IRB.

## Results

### Polymorphisms in *B*. *pertussis* Isolates

Alleles for 48 of *B*. *pertussis* isolates collected from 1989 through 2005 were examined for polymorphism in *ptxA*, *prn*, and *ptxB*. Two alleles of *ptxA* (*ptxA1* and *ptxA2*) were detected in isolates collected from 1989 through 1993 (Table 1). However, all 20 isolates collected during an outbreak in 2005 had *ptxA1* allele. We found 3 alleles of *prn* (*prn1*, *prn2*, and *prn9*). The strains isolated from 1989 through 1993 differed in the *prn* allele, but as observed for the *ptxA*, the 2005 outbreak strains all possessed the *prn2* allele (Table 1).

We also identified a single nucleotide difference (referred to as *ptxB2*) from Tohama I in the Ptx B-subunit gene (*ptxB*) which encodes for the S2 subunit [28]. Surprisingly, even though considerable polymorphisms were observed for the *ptxA* and *prn* alleles, all 48 clinical isolates sequenced in this study had *ptxB2* allele (Table 1). The Chi-squared test was applied to determine whether there was a significant association among the three alleles (*ptxA*, *ptxB*, or *prn*) in the 48 samples. No associations were detected.

We examined the sequence database for other strains with the *ptxB* polymorphisms (Table 2). Both alleles are represented in isolates from the 1950s. In a study of Japanese isolates collected from 1975 through 1996, *ptxB1* was present in 5/12 (42%) strains while *ptxB2* was present in 7/12 (58%) strains [37]. Isolates from the Netherlands and Australia expressed the *ptxB2* allele. Japanese whole cell and acellular vaccines are prepared with Tohama, while strain CS is used for the Chinese acellular vaccine, and both vaccine strains (Japan and China) express the *ptxB1* allele. A mixture of *ptxB* alleles is present for both whole cell and acellular vaccines used in the United States. The strain used for the Connaught whole cell vaccine (TriHIBit) expressed the *ptxB2* allele, while the Lederle adsorbed vaccine strain expressed the *ptxB1* allele. Current FDA-approved acellular vaccines produced by GlaxoSmithKline (Infanrix and Boostrix) express the *ptxB1* allele, while vaccines produced by Sanofi Pasteur (Daptacel and Adacel) express the *ptxB2* allele. Recently, genomes of 345 *B*. *pertussis* strains were sequenced [38,39]. The data suggested that *ptxB2* allele is dominant in the currently circulating strains of *B*. *pertussis* around the world.

### Glycan Recognition

Ptx binds primarily, if not solely, to the glycan residues that decorate the known mammalian receptors [12]. Ptx possesses at least four different glycan binding regions, two on the Ptx-S2 subunit and two on the Ptx-S3 subunit. Sequence alignment (Fig 1B) demonstrates that the *ptxB* polymorphism maps to the wheat germ agglutinin (WGA) homology site [40]. Since binding differences due to polymorphisms in the S2 subunit could be masked by interactions mediated by S3, we assessed binding to purified S2 in the absence of S3. Previous studies have shown the binding subunits S2 and S3 are more stable as dimers in the presence of S4 [12], and we assessed binding of S2/S4 heterodimers using reflectometric interference spectroscopy (RIS), a label-free technique, somewhat analogous to surface plasmon resonance. RIS monitors the thickness of a protein layer bound to a biosensor tip immersed in liquid samples by comparing the shift in the interference patterns of light reflected from a reference layer versus protein bound to the biosensor tip.

**Fig 3:**
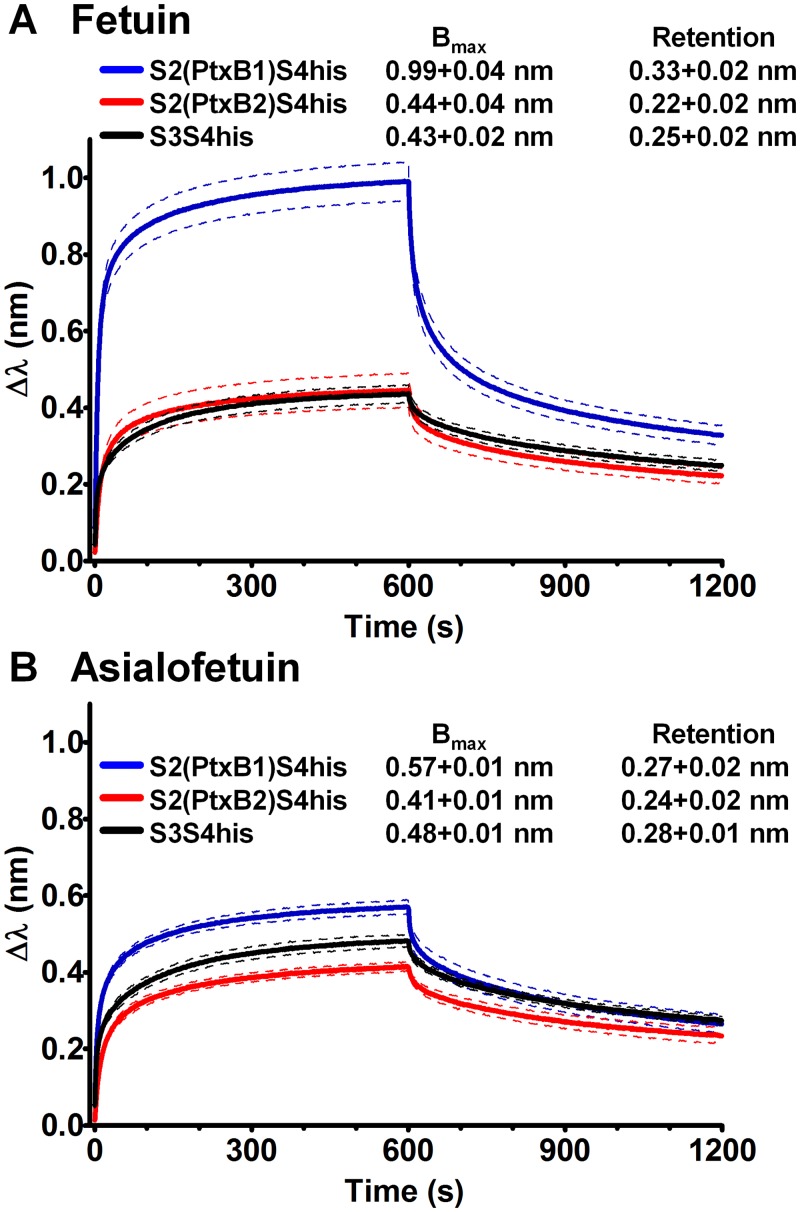
Binding of recombinant dimers to fetuin and asialofetuin. Streptavidin-coated biosensor tips were loaded with biotinylated fetuin or asialofetuin, and the association and dissociation of recombinant dimers (716 nM) was quantified by reflectometric interference spectroscopy. Binding curves represent the mean, solid lines, and standard deviations (SD), dashed lines, of 3 assays. Maximum binding (Bmax) and retention values tabled within the graphs represent the average value for the final 50 second intervals of the association and dissociation phases (respectively) for all three assays. Significantly more S2(PtxB1)S4his was bound (Bmax) and retained (Retention) to fetuin compared to either S2(PtxB2)S4his or S3S4his (Student’s t-test, *P*<0.01). For asialofetuin, while significantly more S2(PtxB1)S4his was bound (Bmax) compared to S2(PtxB2)S4his (Student’s t-test, *P*<0.01), the amounts retained (Retention) by asialofetuin were not different (Student’s t-test, *P* = 0.2).

The bovine serum glycoprotein, fetuin, is a well characterized substrate for Ptx. Fetuin has roughly equal proportions of Neu5Ac-α(2–3)Gal and Neu5Ac-α(2–6)Gal triantennary N-linked glycans [41], where Neu5Ac is the chemical name for sialic acid. Asialofetuin lacks the terminal sialic acid residues. Binding to fetuin and asialofetuin was determined (Fig 3). Similar to previous studies with the Ptx heterodimers [12,15], the kinetic data did not conform to the simple one- or two-site binding models using the ForteBio software or the Biacore BIAevaluation software, but analysis of the dissociation rates suggests multivalent interactions [42]. The S2(PtxB1)S4his form had much faster association and dissociation kinetics resulting in extremely large differences in the maximum binding (B_max_), compared to either S2(PtxB2)S4his or S3S4his. However, very similar binding levels were observed at the end of the dissociation phase (600–1200 seconds) when the tips were incubated in PBS to remove loosely bound toxin (Fig 3A). In contrast, the binding of all three dimers to asialofetuin were similar (Fig 3B). These results are consistent with the hypothesis that the PtxB1 allele encodes a low affinity sialic acid binding site not present in PtxB2. The *ptxB1* allele encodes glycine at amino acid 18 of S2 while the *ptxB2* allele encodes serine at that position. The deeper and wider cleft that forms when glycine is substituted for serine (Fig 1C and 1D) is likely responsible for the faster ON-rate seen in binding to the glycoprotein, fetuin (Fig 3A).

### Cellular Binding

**Fig 4:**
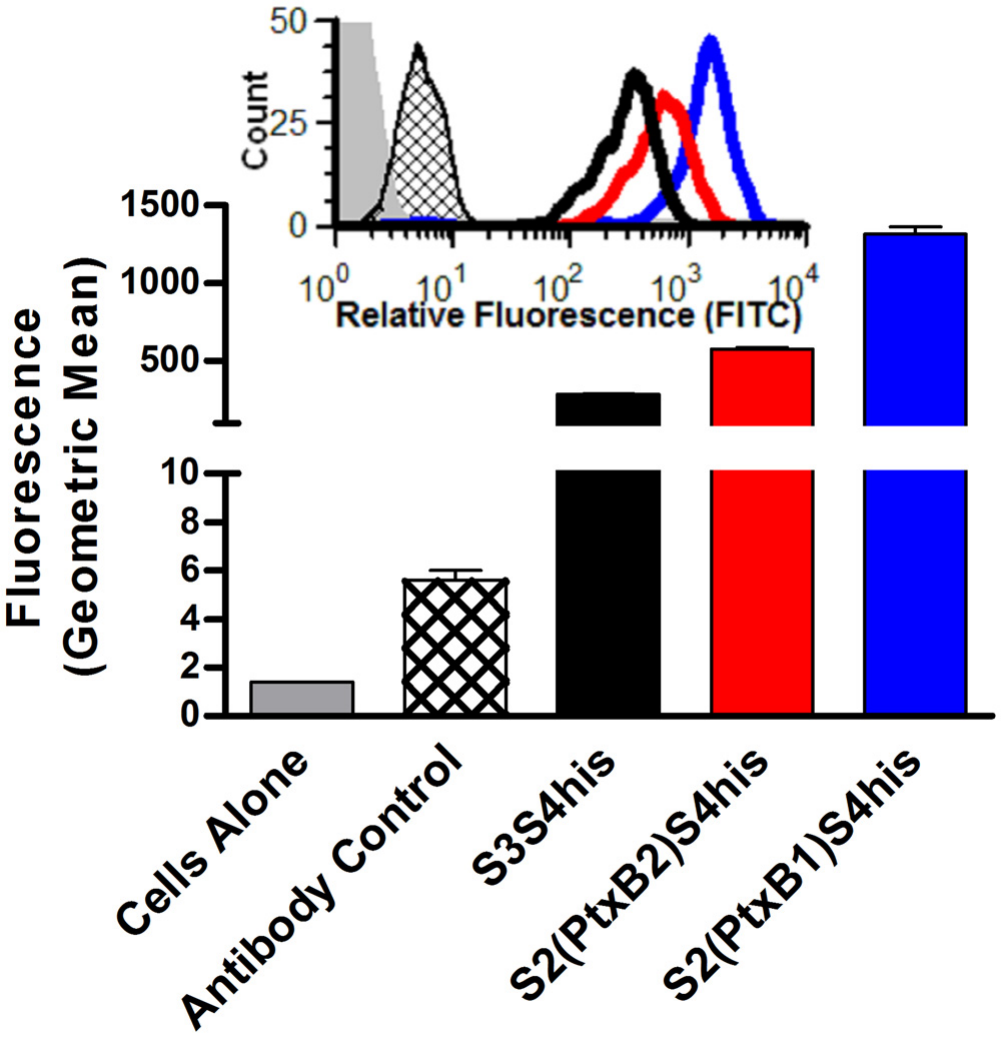
Binding to Jurkat T cells. Recombinant dimers (200 nM) were incubated with Jurkat cells (10^6^) for 1 h at 4°C, and stained with anti-His and labeled secondary antibody (FITC anti-mouse) antibodies. Fluorescence was measured by flow cytometry (inset). Bar graph represents the mean and SD of the population geometric means from 3 assays. Significantly more S2(PtxB1)S4his was bound compared to S2(PtxB2)S4his and significantly more dimer of either form of S2 was bound compared to the S3S4his dimer (Student’s t-test, *P*<0.05).

The ability of recombinant dimers to bind to the human Jurkat T cell line was examined. Purified dimers were incubated with Jurkat cells at 4°C, and stained with anti-His antibody (which recognizes the C-terminal His-tag on S4) and labeled secondary antibody (FITC anti-mouse). Fluorescence was measured by flow cytometry (Fig 4, inset). Significantly more S2(PtxB1)S4his was bound compared to S2(PtxB2)S4his and significantly more dimer of either form of S2 was bound compared to the S3S4his dimer (Fig 4, graph).

### Toxicity

**Fig 5:**
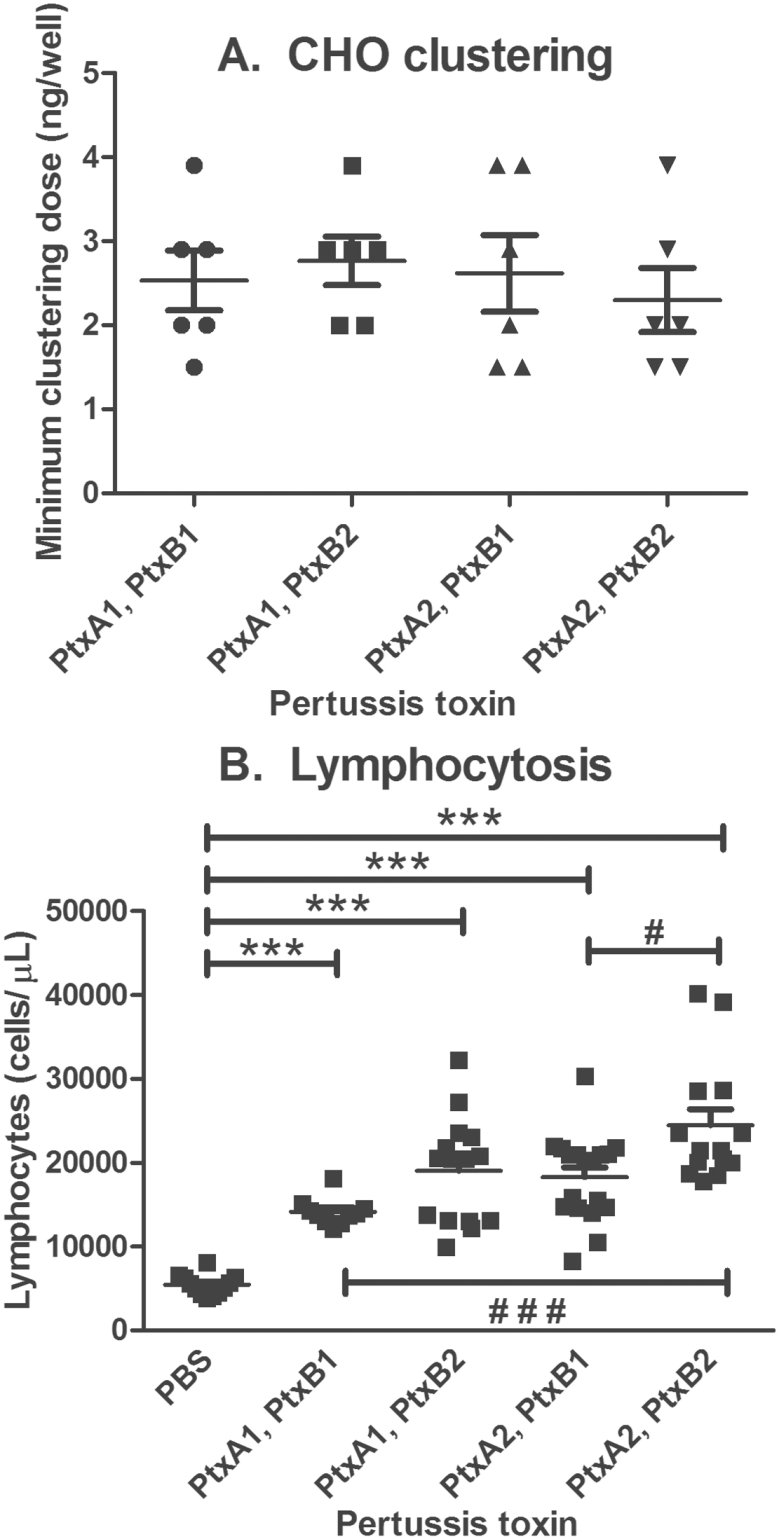
Activities of purified pertussis toxin. **A. CHO clustering activity of purified Ptx holotoxin,** purified from strains VCI (PtxA1, PtxB1), CCHMC1 (PtxA1, PtxB2), Tohama (PtxA2, PtxB1), and KF1 (PtxA2, PtxB2). Vertical lines represent mean and standard error of the mean for six independent experiments. No significant difference was observed (1-way ANOVA, Tukey posttest). **B. Lymphocytosis promoting activity,** of Ptx purified from *B*. *pertussis* strains described above. Vertical lines represent mean and standard error of the mean for data from 10 to 18 mice. All Ptx variants were significantly different from PBS control (1-way ANOVA, Dunnett posttest). In pairwise analysis, PtxA1, PtxB1 and PtxA2, PtxB2, and PtxA2, PtxB1 and PtxA2, PtxB2 were significantly different (1-way ANOVA, Bonferroni’s multiple comparison test). *** or # # #, *P* <0.001; #, *P* <0.05.

Differences in in vitro and in vivo toxicity were examined. Ptx holotoxin was purified from strains expressing different PtxA, and PtxB alleles and examined in vitro for CHO clustering activity. The mean and standard deviations for the minimal CHO cell clustering activity was similar for all of the toxin preparations (Fig 5A), and was not significantly different between the Ptx variants.

Ptx holotoxin is also known to promote elevated lymphocyte cell counts (lymphocytosis) in both humans and mice. We compared the ability of Ptx from the different variants to cause lymphocytosis (Fig 5B). Mice injected with any of the Ptx variants displayed significantly elevated lymphocyte cell counts compared to control mice injected with PBS. In addition, in pairwise comparisons, lymphocyte counts for mice receiving the PtxA2, PtxB2 variant were significantly greater than the PtxA1, PtxB1 varient, and lymphocyte counts for mice receiving the PtxA2, PtxB2 variant were significantly greater than the PtxA2, PtxB1 variant.

### Antigenicity of PtxB Variants

**Fig 6:**
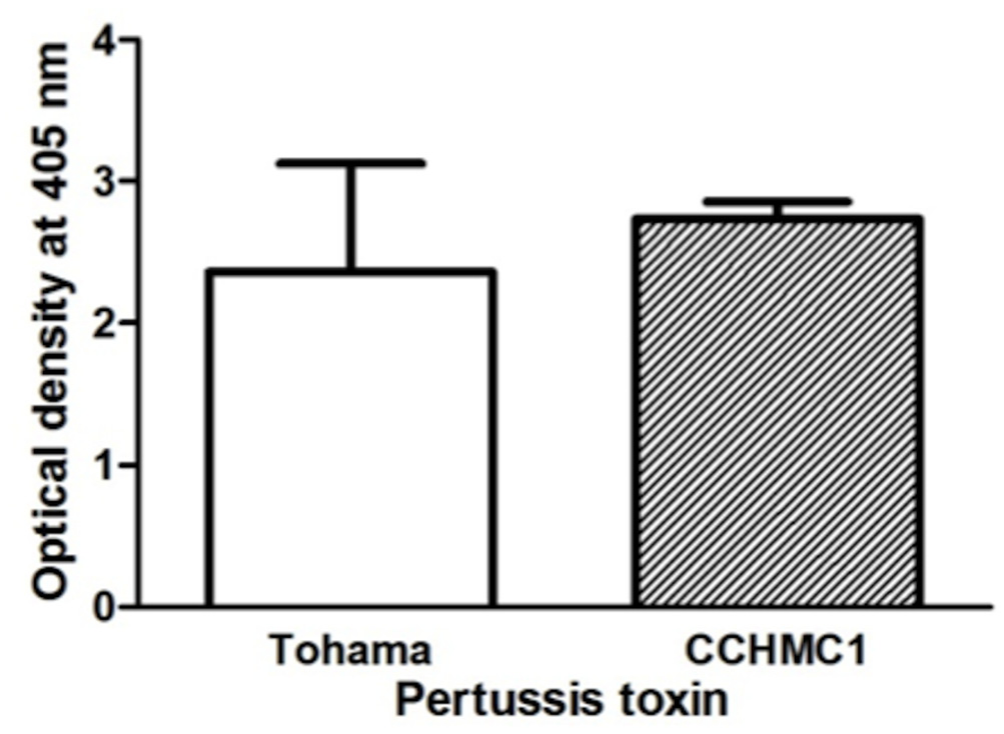
Cross antigenicity between vaccine type pertussis toxin (PtxA2, PtxB1) and non-vaccine type pertussis toxin (PtxA1, PtxB2). Ptx purified from *B*. *pertussis* strain Tohama I (PtxA2, PtxB1), and from strain CCHMC1 (PtxA1, PtxB2) were coated on 96-well plate. ELISA with JNIH-12 antisera prepared from detoxified vaccine type pertussis was performed as described in MATERIALS, AND METHODS. The levels of antibody binding are expressed in terms of mean absorbance. There was no significant difference of antibody binding to each Ptx by Student’s t-test.

Since polymorphisms in the *ptxA* and *prn* genes appear to mediate escape from vaccine detection, we investigated whether the PtxB variants also differ in antigenicity. Anti-Ptx mouse serum (JNIH-12), prepared from mice immunized with detoxified Ptx purified from Tohama I, displayed equivalent recognition for both Ptx forms (Fig 6). These data suggest that detoxified vaccine-type Ptx (PtxA2, PtxB1) could induce antibody that bound to non-vaccine type Ptx (PtxA1, PtxB2).

To determine whether infection or vaccination induces antibodies which recognize the S2/S4 heterodimer, we examined antibody binding to the different S2 polymorphic forms using reflectometric interference spectroscopy. Purified His-tagged S2(PtxB1)S4his and S2(PtxB2)S4his heterodimers were immobilized on Octet sensor tips with anti-His monoclonal antibody, and association and dissociation binding of human immune serum was characterized (Table 3). All of the serum displayed rapid binding in the association phase, similar to the binding seen to fetuin or asialofetuin (Fig 3). However, for most of the serum samples, binding returned to baseline after the dissociation phase (indicated as “-”in Table 3). Weak binding that is easily dissociated is consistent with non-specific binding of serum components to the S2/S4 heterodimers mediated by the S2 glycan binding sites. The lack of binding after the dissociation phase in the antibody studies compared to fetuin or Jurkat cells is likely due to the need to engage multiple binding sites (avidity) for strong binding to occur; weak binding to two glycan binding sites results in tight binding when both sites are bound to the same molecule, or molecules displayed on a solid surface. In the fetuin studies, binding of soluble heterodimer to immobilized fetuin was assessed, whereas in the serum binding studies, binding of soluble serum components to immobilized heterodimer was assessed.

**Table 3:** Characterization of Immune Serum.

Serum Sample^a^	ELISA Units	CHOClustering	PBMCMitogenicity	BindingTo S2/S4
Amvax-1 11	4280	5000	77	+
Biocine-3P 4	4264	5000	77	+
Biocine-3P 13	130	5000	46	-
Biocine-3P 8	77	5000	17	-
CLL-4F 6	552	5000	46	+
CLL-4F 1	64	5000	28	-
APERT 26	3	938	46	-
APERT 37	20	3750	28	-
APERT 40	39	3750	28	-
APERT 43	166	33333	28	-
APERT 47	33	4286	46	-
APERT 48	37	2727	28	-
APERT 50	112	4286	28	-
APERT 52	30	4286	17	-
APERT 54	98	15789	46	-
APERT 55	132	4286	28	-
APERT 57	48	2727	28	-
APERT 60	17	3750	46	-
APERT 63	58	25000	28	-
APERT 68	124	40000	28	-
APERT 72	72	4286	28	-
CCHMC 108	826	54795	128	+
CCHMC 113	463	5263	46	+
CCHMC 120	303	52632	28	-
CCHMC 123	5978	86957	128	+
CCHMC 126	161	4000	46	-
CCHMC 128	89	4000	28	-
CCHMC 131	694	86957	77	-
CCHMC 134	563	86957	46	-
CCHMC 136	107	2740	28	-
CCHMC 139	55	508	46	-
CCHMC 146	4	548	46	-
CCHMC 148	220	1290	77	-

Post vaccination immune sera are indicated by vaccine type (e.g. Amvax-4, Biocine-3P, CLL-4F) or vaccine trial (e.g. APERT). Convalescent sera are indicated by CCHMC. ELISA units, CHO and PBMC neutralization titers were determined in previous studies [32,35,36].

Only six serum samples displayed binding that was greater than the negative control values after the dissociation wash step (Table 3). This strong binding is consistent with the tight binding associated with an antibody response. The Amvax-1, Biocine-3P and CLL-4F 6 vaccines each induced an immune response to S2/S4 dimer in one individual, while none of the 15 subjects in the APERT trial who receive the 3-component GSK vaccine exhibited an immune response to the S2/S4 heterodimers. Three of 12 convalescent sera (CCHMC 108, CCHMC 113 and CCHMC 123) also displayed significant binding to S2/S4 dimers. For the vaccine recipients binding to the S2/S4 dimer correlated best with ELISA values over 500, and not neutralization of CHO clustering or neutralization of PBMC mitogenicity. The CCHMC convalescent samples which bound to the S2/S4 heterodimers also had high ELISA titers, but not all sera with high titers bound to the S2/S4 heterodimers.

**Fig 7:**
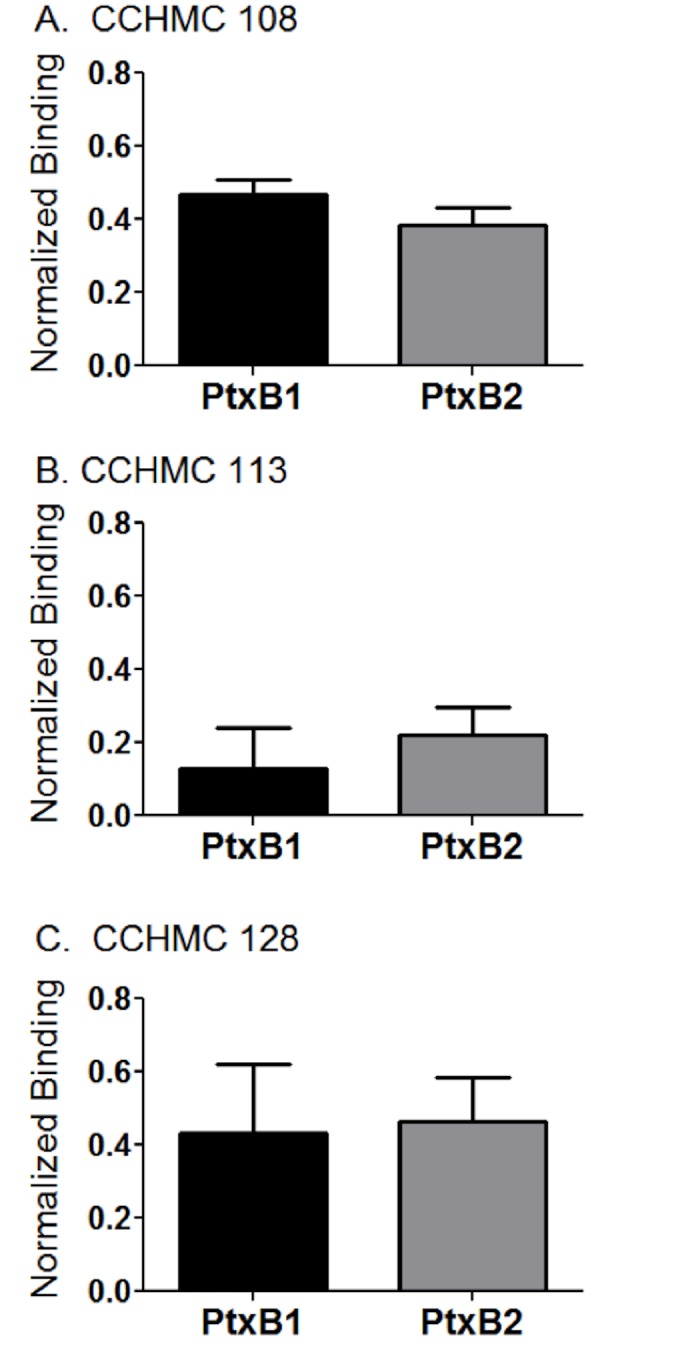
Antibody responses to the S2(PtxB1)S4his or S2(PtxB2)S4his heterodimers as assessed using reflectometric interference. Purified S2/S4 heterodimers were immobilized on HIS2 biosensor tips via the poly-histidine tag on the S4 subunit. Binding to three different convalescent serum samples (diluted 1/100 in PBS) was monitored for 2000 seconds during the association phase. The tips were transferred to wells containing PBS without serum, and dissociation was monitored for 1500 seconds. Three independent repeats were performed; relative binding (mean and standard error of the mean) was determined by dividing the values at 3500 seconds by the value of the heterodimer protein load. No significant differences in binding for the S2(PtxB1)S4his or S2(PtxB2)S4his were observed by Student’s t-test.

The convalescent sera were characterized further to determine if there was preferential binding to S2(PtxB1)S4his or S2(PtxB2)S4his. The samples were tested in three independent binding experiments (Fig 7). To adjust for differences in the amount of heterodimer loaded onto the tip, the post dissociation binding values (3500 seconds) were divided by the value of the load, and the mean and SEM of the three trials is shown (Fig 7). The values for the S2(PtxB1)S4his or S2(PtxB2)S4his heterodimers were not statistically different. This is consistent with the lack of shift in the antigenic index between the two sequences, as predicted by the algorithm of Hopp and Woods [43] http://www.bioinformatics.org/JaMBW/3/1/7/. In all, the S2/S4 heterodimer does not appear to be very immunogenic either following vaccination or infection, and individual who did produce an antibody response to the S2/S4 heterodimer appeared to recognize both polymorphic forms equally well.

## Discussion

We investigated genotypes in clinical isolates collected from 1989 through 2005 in Cincinnati area. About half of the isolates collected from 1989 to and 1993 possessed the vaccine-type genotype (*ptxA2*, *prn1*). However, all of isolates collected in 2005 outbreak possessed the non-vaccine type genotypes in *ptxA1* and *prn2*. A similar trend has been seen in many countries with high vaccine coverage [4], where the dominant genotypes of *ptxA* and *prn* in the circulating strains are different from the vaccine strain. It is widely believed that these changes confer resistance to the heterologous antibody response mediated by vaccine antigens. This is supported by studies in mice where vaccination protects better against challenge with stains expressing the homologous alleles than strains expressing heterologous alleles [8–11].

The *ptxB* alleles appear to be another example of a polymorphism which affects function, but not antigenicity. Polymorphic form, *ptxP3*, maps to the Ptx promoter and encodes for increased expression of the pertussis toxin genes [4]. This allele appears to be replacing other alleles in countries with high vaccination rates, and has been associated with more reported cases, and more severe disease. Another extreme example, that could be either be considered as an antigenic polymorphism or a functional polymorphism is the emergence of virulent *B*. *pertussis* strains that lack expression of Prn [44,45]. Development of pertussis vaccines containing different polymorphic forms of PRN would not increase protection against these variants.

Unlike the *ptxA* allele which alters immunogenicity, but has not been shown to alter protein function [7,8], the S2 B-subunit is poorly immunogenic, and the polymorphism is not associated with altered antibody recognition. However, the PtxB polymorphism is associated with altered activity. PtxB1 bound better to fetuin and T cells than PtxB2. While all holotoxin forms were equally toxic to CHO cells, PtxA2, PtxB2 holotoxin was more toxic to mice than PtxA1, PtxB1, and PtxA2, PtxB1, as evidenced by a greater ability to promote lymphocytosis, suggesting the PtxB2 form could have a greater capacity to cause damage in human disease. It is important to point out we do not know which host cells or cellular receptors are the most important target for advancing the disease process for *B*. *pertussis*. It is possible that PtxB2 allele was selected for its ability to bind more avidly than PtxB1 to the receptors on the target cells which are most important for advancing the disease process. The emergence of Ptx variants which do not rely on antigenic polymorphisms to evade antibody-mediated clearance also suggests reformulating the antigenic composition of Ptx in the acellular pertussis vaccine may not be sufficient to improve efficacy.

## Supporting Information

S1 Fig The Arrive Guidelines Checklist for animal research: reporting in vivo experiments. (PDF)
